# Recent advances in managing tricuspid regurgitation

**DOI:** 10.12688/f1000research.13328.1

**Published:** 2018-03-22

**Authors:** Benedetto Del Forno, Elisabetta Lapenna, Malcom Dalrymple-Hay, Maurizio Taramasso, Alessandro Castiglioni, Ottavio Alfieri, Michele De Bonis

**Affiliations:** 1 IRCCS San Raffaele Hospital, Milan, Italy; 2 Plymouth Hospitals NHS Trust, Department of Cardiac Surgery, Derriford Hospital, Plymouth, UK; 3 UniversitätsSpital Zurich, University Heart Center, Zurich, Switzerland

**Keywords:** Tricuspid valve repair, percutaneous, trans-catheter

## Abstract

Isolated tricuspid valve surgery is usually carried out with very high morbidity and mortality given the complexity of the affected patients. In light of this, trans-catheter tricuspid valve interventions have been emerging as an attractive alternative to surgery over the last few years. Although feasibility has been shown with a number of devices, clinical experience remains preliminary and associated with significant clinical and technical challenges. Here we describe currently available trans-catheter treatment options for severe tricuspid regurgitation implanted in different locations.

## Introduction

Tricuspid valve insufficiency is reported as the most common valvular heart disease, affecting 65 to 85% of the population ^1^; the true prevalence of moderate or severe tricuspid regurgitation (TR) has been estimated at 1.6 million people in the United States ^2^. Despite this prevalence and its known association with mortality ^3^, the tricuspid was considered as the “forgotten valve” until recently for two main reasons. Firstly, symptoms resulting from TR may be uncertain, particularly in the early stages of the disease, leading to a late referral. Secondly, once right ventricular dysfunction develops, the patient is considered end-stage. Moreover, functional TR frequently coexists with left-sided valve disease (LVD), but avoidance of concomitant tricuspid valve repair was accepted based on the incorrect assumption that TR would improve once the primary LVD had been treated ^4^.

When untreated, TR commonly progresses to right ventricular failure, with the associated impact on the patient’s prognosis and quality of life. In the subgroup with advanced disease, the indication for treatment remains more challenging given the complexity of the patients and the significant risk of 30-day mortality ^5^.

Recently, the assessment and management of tricuspid valve disease have developed because of the increasing body of evidence regarding the adverse impact of severe TR and the recent advances in surgical and percutaneous trans-catheter techniques. In the current European guidelines, the indication for surgery for severe TR is now generally accepted (class I). With regard to treatment in lesser degrees of TR, there is a trend for intervention, especially during surgery for left heart valve disease and/or in cases of significant dilatation of the tricuspid annulus (class IIa). In addition, after previous left-sided valve surgery and in the absence of recurrent left-sided valve dysfunction, surgery may be considered in patients with severe TR who are symptomatic or have progressive right ventricular dilatation/dysfunction in the absence of severe right ventricular or left ventricular dysfunction and/or severe pulmonary vascular disease/hypertension ^6^. This more aggressive approach to TR after previous left-sided valve surgery may result in dealing with fewer end-stage patients who could benefit from the treatment.

Despite recent progress in the understanding of this complex pathology and the attempt to treat these patients at the earliest possible stage, the majority remain a high-risk population affected by severe TR, right ventricular dysfunction, and multiple comorbidities. In view of the above, different trans-catheter tricuspid valve therapies are emerging.

## Challenging anatomical features for trans-catheter therapies

The anatomy of the tricuspid valve apparatus appears to be more complex than the mitral valve. It is variable in terms of number, size, and attachment of the papillary muscle and, in contrast to the mitral valve, tricuspid valve chordae may attach directly to the right ventricular endocardium.

The tricuspid annulus is an ellipsoid nonplanar structure with a saddle-shaped conformation that becomes more circular as it dilates in an anterior-posterior direction in response to right ventricular enlargement, which is also a dynamic structure that modifies its dimension and shape during the cardiac cycle ^7^.

Several aspects represent a challenge for percutaneous therapy. The tricuspid orifice is larger, more triangular, and more fragile than the mitral one; in addition, the lack of calcium observed in secondary TR represents an anchorage-complicating factor.

The tricuspid annulus has significant anatomical relationships with the right coronary artery, the coronary sinus, the aortic valve, and the atrio-ventricular node. All of these structures can be damaged during trans-catheter procedures.

The angulation between both venae cavae and the tricuspid annulus should be taken into consideration in the conception of a delivery system for percutaneous approach; alternatively, if considering a trans-apical approach, the thin apical wall and the presence of multiple chordae render this approach very difficult ^8^.

Finally, in a considerable portion of patients, the secondary TR is due to the presence of pacemaker or defibrillator leads ^9^ that represent a limitation in applying percutaneous techniques.

## Multi-modal imaging to plan trans-catheter therapies

Quantification of the severity of TR has been well described by the European Association of Echocardiography guidelines, clearly defining qualitative and quantitative parameters ^10^. Moreover, considering the last guidelines of the European Society of Cardiology, the measurement of the septo-lateral dimension of the tricuspid annulus to identify significant annular dilatation (diastolic dimension ≥40 mm or >21 mm/m ^2^) is important, as it supports intervention ^11^.

To perform a first evaluation, a trans-thoracic echocardiogram is enough, but, to correctly plan a percutaneous intervention, a multi-modal imaging approach is mandatory. Three-dimensional trans-esophageal echocardiogram has significantly improved the accuracy of tricuspid valve apparatus imaging, giving adjunctive information on its anatomic components and highlighting the lesions liable for the mechanism of regurgitation. A standardization of imaging projections has been proposed by Lang *et al*. ^12^ to obtain an en face view of the tricuspid valve, improving communication with the interventionist.

Pre-procedural planning using multi-detector computed tomography (MDCT) provides many details regarding tricuspid annulus measurement, the relationship with the surrounding structures, and good-quality motion-free images of the right ventricular outflow tracts ^13^. Furthermore, MDCT is used to access routes for percutaneous procedures.

Applying this multi-modal imaging approach is crucial to obtain essential information to correctly choose the proper trans-catheter technique.

## Trans-catheter therapies for tricuspid regurgitation

Given the considerable surgical risk of tricuspid valve surgery for severe TR, especially in the reoperation scenario, there has been a progressive development of percutaneous trans-catheter techniques in the last few years. Anatomical aspects, such as the non-planar and large tricuspid annulus, absence of calcium, right ventricular geometry, and approximation of critical surrounding structures, have to be taken into consideration and make this procedure very challenging ^14^.

The Gate™ Tricuspid Valved Stent (Navigate Cardiac Structure Inc.) is the first prosthesis implanted in the tricuspid position using a trans-jugular or trans-atrial mode of access and has been implanted successfully in two patients ^15^. As an alternative to trans-catheter tricuspid valve implantation, four kinds of percutaneous therapies have recently emerged to treat severe tricuspid valve regurgitation: trans-catheter heterotopic implant of valve into the vena cava, trans-catheter annuloplasty, trans-catheter edge-to-edge repair, and trans-catheter implant of a device dedicated to improve leaflet coaptation.

### Heterotopic caval trans-catheter valve implantation

Caval valve implantation (CAVI) has been proposed to address the reflux of severe TR into both venae with the purpose of effectively improving the symptoms of right ventricular failure. The main anatomical challenges in applying this specific technique is the large and variable diameter of the inferior and superior vena cava, especially in the presence of chronic severe TR, and the length of the landing zone with respect to the inferior vena cava (IVC) and the closeness to the hepatic veins.

Clinical experience started in 2011, with the first reported compassionate case of CAVI using two custom-made self-expandable valves ^16^. Since then, compassionate cases confirmed the feasibility of this technique and the immediate hemodynamic improvement ^17^. In light of this, two valve prototypes, the self-expandable TricValve (P+F Products + Features Vertriebs GmbH, Vienna, Austria, in cooperation with Braile Biomedica, São José do Rio Preto, Brazil) and the balloon-expandable Edwards valve (Edwards Lifesciences, Irvine, CA, USA), have been tested in proof-of-concept trials.

The TricValve consists of two self-expandable bioprosthetic valves covering sizes from 28 to 43 mm for both caval veins. This device does not require pre-stenting of the cavae to permit its implant. The prosthetic valve conceived for the IVC has the upper segment protruding into the right atrium to avoid any dangerous interaction with hepatic veins. The device designed for the superior vena cava is mounted on a funnel-shaped stent frame to allow its release at the level of the cavo–atrial junction. The procedure is guided by fluoroscopy, and echocardiography is used for post-interventional control.

Concerning the heterotopic caval implantation of 29 mm balloon-expandable Edwards SAPIEN XT or SAPIEN 3 valves, it has been performed off-label for patients at prohibitive surgery risk. To consider a safe implantation of this prosthesis, a self-expandable stent was implanted at the level of the cavo–atrial junction to create a landing zone for valve anchoring. Ten compassionate cases have been performed, but, although the implant was successfully achieved in all patients without peri-procedural complications, the 9-month follow-up mortality was 90% ^18^.

### Trans-catheter tricuspid valve annuloplasty

Severe functional TR is due to a significant annular dilatation, and that is why surgical annuloplasty is the first procedure of choice in treating this condition. Applying this principle to the percutaneous techniques results in several complexities. Unlike its left counterpart, the tricuspid annulus is not prominent or fibrous and for this reason does not ensure proper stability for safely anchoring some devices. Moreover, the highly individual shape of the valve adds complexity. Finally, several crucial structures are closely related to the tricuspid annulus and are at risk of injury.

The Trialign device (Mitralign, Inc. Tewksbury, MA, USA) mimics the Kay surgical procedure consisting of plication of the tricuspid annulus at the level of the posterior leaflet ^19^. The operator performs a trans-catheter bicuspidization of the tricuspid valve through a trans-venous jugular approach, implanting one or multiple pairs of pledgets. Once implanted, the pledgeted sutures are brought together to obtain plication of the annulus. The first-in-human experience with this system has been reported, demonstrating the feasibility of this procedure and resulting in a reduction of the tricuspid annular dimensions ^20^.

The safety and feasibility of this device has been tested in the SCOUT I feasibility trial, which included 15 patients with severe TR who underwent tricuspid annuloplasty with the Trialign. The device was successfully implanted in all cases, with only one procedural complication (new right coronary stenosis requiring coronary stenting). At 30-day follow-up, survival was 100%, with a technical success rate of 80% (three single pledget dehiscence). No major adverse events were observed. A significant reduction in the tricuspid annular area, as well as an improvement in functional class and quality of life, was maintained for up to 6 months ^21^.

The TriCinch system (4Tech Cardio, Galway, Ireland) is a percutaneous annuloplasty device designed to cinch the tricuspid annulus, improving leaflet coaptation and reducing TR ^22^. Through a transfemoral mode of access, a corkscrew element is implanted in the anterior tricuspid annulus. Once the corkscrew is secured, the system is retrieved and a self-expandable nitinol stent is introduced over the wire and coupled to the implant. The whole system is then tensioned to reshape the tricuspid valve and to increase the leaflet coaptation under live echo guidance. Finally, the stent is deployed in the IVC to maintain the tension applied. The first-in-human implantation of this device has been reported ^23^. The device is currently being evaluated in an ongoing feasibility trial, the PREVENT study (Percutaneous Treatment of Tricuspid Valve Regurgitation with the TriCinch System; NCT02098200) with the purpose of demonstrating immediate and post-operative safety and efficacy in TR reduction (≥1 grade) in 24 patients. A second-generation device with improved durability is currently under preliminary clinical evaluation. The first-in-man patient has been recently successfully treated ^24^.

**Figure 1.**
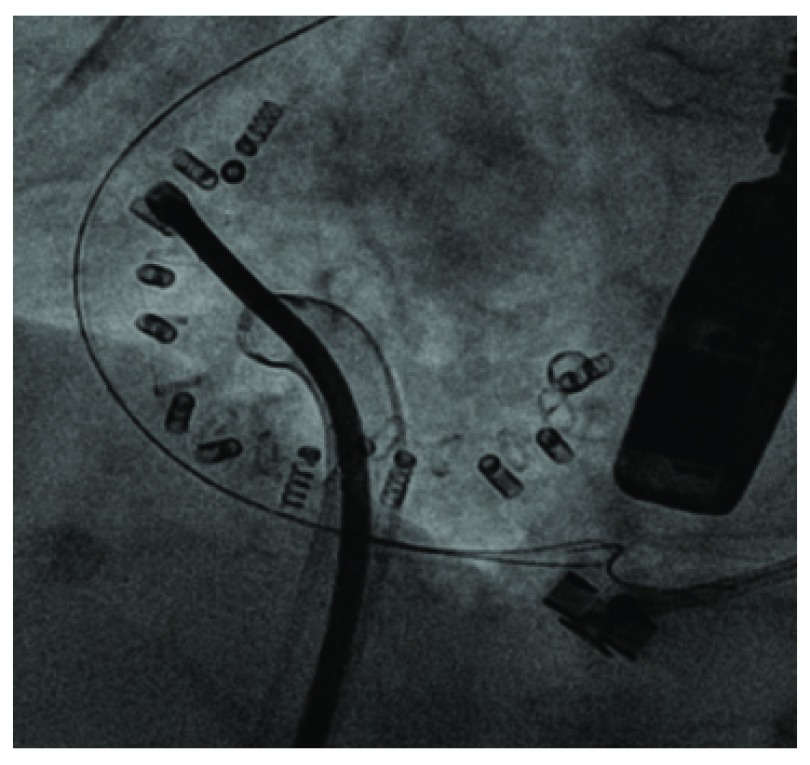
Intra-procedural imaging of the Cardioband system (Edwards Lifescience, Irvine, CA, USA).

The Cardioband system (Edwards Lifescience, Irvine, CA, USA) is a suture-less and adjustable surgical-like Dacron band that mimics the effects of undersized annuloplasty ( Figure 1). It consists of a three-step approach: implant deployment, anchor implantation, and size adjustment. A percutaneous transfemoral approach is used and the device is implanted on the atrial side of the tricuspid annulus by means of multiple anchor elements. After the implant, the size of the device can be reduced in a reversible fashion in order to reduce the annular dimension and increase leaflet coaptation. Preliminary results from the early feasibility trial in the tricuspid position have been recently presented ^25^. Among 20 patients with severe functional TR treated so far, a 27% reduction of septo-lateral tricuspid annular dimension has been reported. At 30 days, core-lab adjudicated data (available for a minority of patients) showed a reduction of TR, with a significant improvement in symptoms and quality of life.

The Millipede system (Millipede, LLC, Ann Arbor, MI, USA) is a fully repositionable and retrievable complete ring, which can be implanted surgically or in a trans-catheter fashion on the atrial side of the native tricuspid annulus in order to restore its shape and diameter. So far, only two patients have received a tricuspid implant under direct surgical vision, showing tricuspid diameter reductions of 42–45%, with no post-procedural residual TR ^26^.

### Trans-catheter edge-to-edge repair

**Figure 2.**
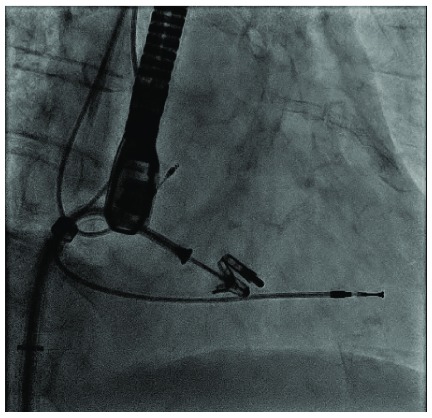
Intra-procedural imaging of the MitraClip® device (Abbott Vascular, Santa Clara, CA, USA).

The MitraClip® device (Abbott Vascular, Santa Clara, CA, USA) consists of a 4 mm wide cobalt-chromium, polyester-covered implant with two arms that are opened and closed by control mechanisms on the clip delivery system ( Figure 2). During the last few years, the MitraClip® has become an attractive trans-catheter alternative to treat selected patients affected by severe mitral regurgitation and recently has been used successfully for treating severe TR in very high-risk patients ^27^. The transfemoral access has become the preferred route of access today. Nickenig *et al*. recently reported promising 30-day outcomes in a series of 64 high-risk patients with severe functional TR treated with tricuspid clipping. A significant reduction in TR and clinical improvements were observed ^28^.

### Coaptation device

The FORMA Repair System (Edwards Lifesciences, Irvine, CA, USA) acts as a coaptation device with the purpose of reducing the TR grade in patients with severe TR. It is composed of a spacer, which is positioned into the regurgitant orifice, creating a platform for the native leaflet to coapt, and a rail anchored at the right ventricular apex, which ensures device fixation. The spacer consists of a foam-filled polymer balloon available in two sizes (12 and 15 mm). Introduced through venous subclavian access, after the final positioning, it is proximally locked at the subclavian region.

To date, seven patients treated with this device have been reported ^29^. Initial first-in-man experience shows that the device was correctly implanted in all patients without any complication. There was no in-hospital mortality, and all of the treated patients completed 30-day follow-up without further evidence of device-related complication. A reduction in the severity of TR was observed in all patients as assessed by transthoracic echocardiography. This change in TR was associated with a significant improvement in New York Heart Association (NYHA) functional classification, accompanied by relevant reductions in peripheral edema in all patients. The clinical outcomes (up to 1 year) of the first 18 patients have been reported recently ^30^. Acute TR reduction of at least one grade was achieved in 89% patients, with an extremely low incidence of adverse events. Significant improvements in functional status and reverse remodeling of the RV were observed at follow-up. An early feasibility trial is currently ongoing in the United States (NCT02471807).

## Results

All of the above-mentioned percutaneous techniques have emerged during the last few years with the purpose of treating patients with severe symptomatic functional TR, who conventionally represent a high-risk surgical population. Most of the patients percutaneously treated today are affected by end-stage heart failure, with right ventricular dysfunction and severe comorbidities, and these therapeutic options are offered in a compassionate treatment scenario. In light of this, assessing the real clinical benefits of trans-catheter tricuspid valve therapies remains extremely difficult, as well as identifying which patients may benefit from an interventional treatment.

In order to address these issues, the first international registry for trans-catheter tricuspid valve therapies (the international multicenter TriValve registry) was established to collect data regarding patients undergoing tricuspid valve interventions with currently available devices ^31^. To date, the TriValve registry has data from 106 patients with severe TR from 11 cardiac centers. Functional TR was present in 95.2% of patients, with a mean tricuspid annulus of 45.4 ± 11 mm. In 56.3% of patients, there was right ventricular dysfunction defined as TAPSE <17 mm and 95% of the entire cohort were in NYHA functional class III to IV. A total of 35% of patients had prior left heart valve intervention.

A total of 55% of patients (n = 58) were treated by MitraClip, 16% by Trialign (n = 17), 14% by TriCinch (n = 15), 7% by FORMA (n = 7), 5% by Cardioband (n = 5), and 3% of patients (n = 3) underwent CAVI. No differences in terms of EURO-Score II and degree of right ventricular dysfunction were observed between the patients treated with the different techniques.

Procedural success was achieved in 62% of cases. At 30-day follow-up, all-cause mortality was 3.7%, with an overall incidence of major adverse cardiac and cerebrovascular events of 26%; 58% of patients were NYHA functional class I or II at 30 days. The dosage of diuretics was significantly reduced at follow-up compared to baseline, as was the degree of TR (≤2+ in 49% of patients).

These initial results showed that the multiple percutaneous devices available for the treatment of TR are being utilized in patients at high surgical risk with early clinical efficacy, but further studies are necessary to better define optimal patient selection, timing of intervention, disease severity, and device efficacy ^31^.

## Reporting outcomes after trans-catheter tricuspid valve therapies: towards T-VARC

The analysis of preliminary results from the early feasibility trials with the different devices raises the issue of how to report outcomes after trans-catheter tricuspid valve therapies. Patients consistently report improvements in quality of life measures despite only modest reductions in TR, according to the conventional definitions. Given the discordance between TR reduction and clinical improvement, it is evident that we cannot use the definitions of procedural success for the tricuspid valve that are used for aortic and mitral valves. This observation raises the need to define appropriate outcomes in this patient population and develop standard definitions on how to evaluate and report outcomes.
